# Dicarbonyl Stress and Glyoxalase-1 in Skeletal Muscle: Implications for Insulin Resistance and Type 2 Diabetes

**DOI:** 10.3389/fcvm.2018.00117

**Published:** 2018-09-10

**Authors:** Jacob T. Mey, Jacob M. Haus

**Affiliations:** ^1^Department of Pathobiology, Cleveland Clinic, Cleveland, OH, United States; ^2^School of Kinesiology, University of Michigan, Ann Arbor, MI, United States

**Keywords:** type 2 diabetes, insulin resistance, aerobic exercise, methylglyoxal, advanced glycation endproducts

## Abstract

Glyoxalase-1 (GLO1) is a ubiquitously expressed cytosolic protein which plays a role in the natural maintenance of cellular health and is abundantly expressed in human skeletal muscle. A consequence of reduced GLO1 protein expression is cellular dicarbonyl stress, which is elevated in obesity, insulin resistance and type 2 diabetes (T2DM). Both *in vitro* and pre-clinical models suggest dicarbonyl stress *per se* induces insulin resistance and is prevented by GLO1 overexpression, implicating a potential role for GLO1 therapy in insulin resistance and type 2 diabetes (T2DM). Recent work has identified the therapeutic potential of novel natural agents as a GLO1 inducer, which resulted in improved whole-body metabolism in obese adults. Given skeletal muscle is a major contributor to whole-body glucose, lipid, and protein metabolism, such GLO1 inducers may act, in part, through mechanisms in skeletal muscle. Currently, investigations examining the specificity of dicarbonyl stress and GLO1 biology in human skeletal muscle are lacking. Recent work from our lab indicates that dysregulation of GLO1 in skeletal muscle may underlie human insulin resistance and that exercise training may impart therapeutic benefits. This minireview will summarize the existing human literature examining skeletal muscle GLO1 and highlight the emerging therapeutic concepts for GLO1 gain-of-function in conditions such as insulin resistance and cardiometabolic disease.

## Introduction

Despite decades of coordinated efforts, T2DM remains a serious public health issue. In the United States alone, over 30 million adults are suffering from T2DM (1) and ~86 million are pre-diabetic and without intervention, may develop overt T2DM as well (2). T2DM is preceded by progressive skeletal muscle insulin resistance (3). Although the etiology and pathology of skeletal muscle insulin resistance and progression to T2DM remain multifactorial, emerging research implicates skeletal muscle dicarbonyl stress as a causative factor. Dicarbonyl stress is described as abnormally high concentrations of methylglyoxal (MG; a highly reactive dicarbonyl) which is elevated in diabetes (4). Methylglyoxal (MG) is a potent intracellular glycating agent that forms advanced glycation endproducts (AGEs). Formed spontaneously from 3-carbon glycolytic intermediates, MG rapidly glycates proteins and nucleotides, damage mitochondria and directly increase reactive oxygen species (ROS) production; thus, inducing a pro-oxidant state and senescent-like condition (4). Drug therapies developed to combat dicarbonyl stress that function as “MG scavengers” have had little clinical success; alternate therapies are being developed to utilize the natural defense against dicarbonyl stress, the glyoxalase enzymatic defense system.

The glyoxalase enzymatic defense system prevents dicarbonyl stress and is controlled by the protein expression and enzymatic activity of glyoxalase 1 (GLO1). GLO1 is a ubiquitously expressed enzyme that directly detoxifies MG, thus, mitigating dicarbonyl stress. However, under pathological conditions like insulin resistance and T2DM, GLO1 is reduced while MG generation is increased, creating an environment in which dicarbonyl stress persists. These phenomenon are well known to contribute to complications associated with diabetes such as nephropathy (5), neuropathy (6), and retinopathy (7). To date, much of the MG and GLO1 research has been directed at insulin-independent tissues (kidney, nervous, retina, respectively) whereas these tissues do not require insulin action for glucose disposal through facilitated glucose uptake via GLUT4 vesicles. However, recent evidence suggests the dysregulation of the MG-GLO1 axis extends to the highly metabolic skeletal muscle tissue and may play a causative role in the development of insulin resistance and overt T2DM.

We have recently published human data describing a dysregulation of dicarbonyl stress concomitant with reduced GLO1 protein expression in skeletal muscle of individuals with T2DM (8). Further, *in vitro*, pre-clinical evidence shows GLO1 overexpression protects against insulin resistance and T2DM. Clinically, two promising human therapies have arisen: nutraceutical GLO1 inducer therapy and aerobic exercise training. This minireview will summarize the physiologic impact of dicarbonyl stress and GLO1 in skeletal muscle metabolism and highlight the emerging therapeutic concepts for combating dicarbonyl stress.

## Importance of skeletal muscle

Skeletal muscles are one of the most metabolically important tissues in the body. Striated muscle tissue accounts for >33% total body mass (9), >40% total body protein (10) and plays a major role in glucose disposal, accounting for >80% of insulin stimulated glucose uptake (11). Skeletal muscle insulin resistance is defined as reduced glucose uptake in response to insulin and is implicated in the pathogenesis of many diseases, most prominently T2DM.

Although multiple etiologies of T2DM exist, including ß-cell dysfunction, dysregulated fatty acid metabolism, and hepatic insulin resistance (12), the primary defect remains skeletal muscle insulin resistance (13–15), often as a consequence of a sedentary lifestyle combined with excessive kilocalorie consumption (16). Hyperinsulinemic-euglycemic clamp data has shown that individuals with T2DM have a >50% reduction in insulin stimulated glucose disposal compared to healthy individuals (17), primarily due to reductions in skeletal muscle glucose uptake.

Despite reductions in glucose disposal, even insulin-resistant skeletal muscle is exposed to a significant amount of glucose flux. For example, glucose disposal rates of insulin-resistant individuals measured during the hyperinsulinemic-euglycemic clamp, although reduced compared to insulin-sensitive individuals, remain at ~2–3 mg of glucose/kg body weight/minute (18). Improper handling of this glucose flux can have deleterious effects (19) which occur prior to the onset of overt T2DM (20); for example, the generation of methylglyoxal (MG) and dicarbonyl stress.

## MG and dicarbonyl stress in skeletal muscle and insulin resistance

Methylglyoxal is a 3-carbon, highly reactive α-oxoaldehyde, or “dicarbonyl” that modifies proteins via covalent bonding. MG-modification may induce protein changes including, loss of side chain charge, alter structure and function (21, 22), signal proteolytic degradation (23) or AGE formation (24). MG-modifications are commonly directed at arginine residues and form methylglyoxal-derived hydroimidazolone adducts (MG-H1) (25). MG-H1 adducts are particularly deleterious due to the high probability of arginine residues to be located at functional sites of proteins (26). Proteins with altered function after MG-modification are known as the dicarbonyl proteome, which is an area of active research investigation. MG and MG-H1 are elevated in plasma and tissues of individuals with T2DM and are well-known to contribute to diabetic complications such as diabetic nephropathy (5), neuropathy (6), retinopathy (7), and early cardiovascular disease (27–29).

The majority of MG formation is spontaneously produced *in vivo* during glycolysis from the 3-carbon glycolytic intermediates, dihydroxy-acetone-phosphate (DHAP) and glyceraldehyde-3-phosphate (G3P). Spontaneous MG formation from DHAP and G3P occurs at a constant rate of ~0.05–0.1% of glycolytic flux (30). In T2DM, a dysregulation of glycolysis leads to a buildup of 3-carbon intermediates and subsequent increased generation of MG (28). Alternatively, MG can form from lipid peroxidation mechanisms, known to be exacerbated with oxidative stress, a common characteristic of insulin resistance and T2DM (13). MG induces pathways known to contribute to insulin resistance including: (1) oxidative stress caused by damage to mitochondria (31) and mitochondrial DNA (32), (2) generation of AGEs [for more information on the independent effects of AGEs in diabetes and diabetic complications, the reader is directed to a recent review by Brings et al. (33)] and (3) inflammation mediated through the Receptor for Advanced Glycation Endproducts (RAGE) signaling (34–36). RAGE is highly expressed in skeletal muscle (37, 38) and upon binding of a RAGE-ligands (i.e., MG-H1) a well characterized inflammatory signaling cascade ensues (39–41). Further, *in vitro* experiments in L6 myotubes treated with MG or MG-modified proteins, inhibit insulin-stimulated glucose uptake via impaired phosphorylation of phosphatidylinositol-4,5-bisphosphate 3-kinase (P13K) and extracellular-signal-regulated kinase 1 and 2 (ERK1/2) (42–44). This induction of insulin resistance was independent of MG-generated oxidative stress and likely due to direct binding of MG to the important insulin signaling protein, IRS-1.

## The glyoxalase enzymatic defense system

The biological natural defense against dicarbonyl stress is the glyoxalase enzymatic defense system which detoxifies MG by converting it to a stable byproduct (D-Lactate). This enzymatic system is controlled by the protein expression and enzymatic activity of GLO1. GLO1 is ubiquitously expressed in all cells and catalyzes the first step in the glutathione-dependent sequestration of MG at an astounding (>99%) efficiency (45). For further information on the glyoxalase detoxification mechanism, we refer the reader to literature and reviews by Thornalley et al. (46–49). GLO1 is essential to protecting cells from dicarbonyl stress and is highly expressed in skeletal muscle (50–55).

Human, animal and *in vitro* studies describe GLO1 as the primary regulator of dicarbonyl stress in tissues (48), including skeletal muscle (56). The importance of GLO1 in preventing dicarbonyl stress in human physiology is evidenced by its ubiquitously expressed nature, as it is present in the cytosol of all cells, and its abundance, remaining in the top 13% of human proteins with a concentration of ~0.2 μg of GLO1 per 1 milligram of human tissue (57). Additionally, a rare mutation in human *GLO1* gene that produces a non-functional GLO1 protein is embryonically lethal due to an inability to prevent dicarbonyl stress (58). Gene deletion of GLO1 is also embryonically lethal in mice (59) whereas in *Drosophila*, GLO1 knockout animals displayed increased MG and recapitulated the progression of T2DM with obesity, insulin resistance and hyperglycemia (60). Mechanistic evidence from *in vitro* studies using the GLO1 inhibitor, *Staltil*, resulted in loss of GLO1 function and MG accumulation in a variety of tissues including skeletal muscle (61, 62). Further, silencing of GLO1 also increases MG and contributes to the development of multiple disease conditions (63).

In metabolically healthy muscle cells, transient increases in MG stimulate GLO1 protein expression resulting in efficient detoxification of MG despite increased MG flux (64). However, in metabolically compromised muscle (as seen in insulin resistance and T2DM), reduced efficiency of the glyoxalase system leads to MG accumulation (8, 61, 62). This increase in intracellular MG results in inhibition of insulin signaling (42–44, 65), damage to mitochondria (31) and mitochondrial DNA (32) increased ROS production (35), inflammation related to the accumulation of MG-H1 (34) and structural changes to skeletal muscle proteins (56). The causes of reduced GLO1 in skeletal muscle is largely unknown however, we recently described concomitant reductions in GLO1 and NRF2 protein expression, the transcriptional regulator of GLO1 in T2DM subjects (8). NRF2 is highly sensitive to REDOX perturbations and hypoxia suggesting that adequate tissue blood flow may also play a role in GLO1 biology. Recent murine data has also provided evidence for reduced GLO1 protein expression with diet induced obesity (66).

In contrast to the theory that MG is causative to skeletal muscle insulin resistance, Gawlowski et al. (67) recorded *increased* glucose uptake in L6 myoblasts with siRNA GLO1 knockdown and reported altered GLUT4 trafficking mediated by MG accumulation. This discrepancy is likely due, in part, to MG-mediated oxidative stress. MG is known to increase mitochondrial reactive oxygen species (ROS) generation, which increases glucose uptake and stimulates GLUT4 translocation in muscle cells (68, 69). The increased glucose uptake observed by Gawlowski et al. may be an artifact of mitochondrial ROS rather than from the MG accumulation *per se*, which would agree with the findings of others (42–44). Future studies should continue to differentiate the effects of MG-mediated ROS production and independent effects of MG.

Although GLO1 is highly expressed in skeletal muscle tissue in humans (50–55), information on GLO1 protein expression and activity as it pertains to insulin resistance and T2DM is lacking. To date, there are limited investigations in the area of GLO1 and skeletal muscle. Table 1 highlights these studies and their major findings. In muscular dystrophy [a disease characterized by skeletal muscle insulin resistance (70)] GLO1 protein was reduced. Similarly, observations from our lab show GLO1 protein expression and enzyme activity is reduced in obese insulin resistant individuals and GLO1 activity was strongly correlated with insulin sensitivity, and VO_2max_ but inversely correlated with chronological age and percentage body fat (73). Moreover, we recently reported marked reduction in skeletal muscle GLO1 protein expression in individuals with T2DM compared to lean healthy control subjects (8). Of note, shotgun proteomic approaches have yielded conflicting reports with regard to GLO1 (53–55, 72). Despite the current state of the literature, we cannot differentiate independent effects of obesity, insulin resistance or clinical T2DM on GLO1 protein expression or enzymatic activity; nor can it be determined if reductions in GLO1 are causative or a consequence of metabolic pathologies. Future studies should utilize clinical-translational approaches to better elucidate the role of skeletal muscle GLO1 protein expression and enzymatic activity in metabolic pathologies.

**Table 1:** Studies Investigating Glo1 In Human Skeletal Muscle.

**Publication**	**Measurement**	**Population**	**Intervention**	**Timepoint**	**Outcome**
Kar and Pearson (70)	GLO1 activity	Muscular dystrophy (*n* = 24), controls (*n* = 6)	n/a	Basal	Reduced in muscular dystrophy
Haralambie and Mossinger (50)	GLO1 activity	LHC, trained cyclists (*n* = 7 each)	n/a	Basal	Trend of 21% increase in trained cyclists
Radom-Aizik et al. (71)	Broad-scale genome profiling (gene microarray)	Elderly, sedentary men (*n* = 6)	12-week aerobic exercise training	Pre/post exercise (chronic)	Gene expression increased after AE
Hwang et al. (53)	Broad-scale proteomic profiling (HPLC-MS/MS)	LHC, Obese, T2DM (*n* = 8 each)	n/a	Basal	No significant difference between groups
Hussey et al. (72)	Broad-scale proteomic profiling (HPLC-MS/MS)	T2DM (*n* = 6), controls (*n* = 6)	4 weeks, 5 days/week, aerobic training and HIIT, T2DM only	Pre/post exercise (chronic)	No effect of T2DM, decreased post exercise in T2DM
Hoffman et al. (51)	Broad-scale proteomic profiling (TMT-MS/MS)	LHC (*n* = 4)	Single bout (high-intensity cycle exercise)	Pre/post exercise (acute)	Nonsignificant 21% increase
Mey et al. (8)	Qualitative proteomic characterization (Western Blot)	LHC (*n* = 10), T2DM (*n* = 5)	Hyperinsulinemic-euglycemic clamp	Basal and Insulin	Reduced in T2DM, no effect of insulin

*The current state of the literature suggests GLO1 protein expression and enzymatic activity is reduced in skeletal muscle in insulin resistance and T2DM. Further, aerobic exercise may provide a therapeutic effect by increasing skeletal muscle GLO1. Additional research is warranted to corroborate these findings. LHC, lean healthy control subjects; T2DM, individuals with Type 2 Diabetes; GLO1, glyoxlase-1; AE, aerobic exercise; HPLC-MS/MS, high performance liquid chromatography tandem mass spectrometry; TMT, tandem mass tags; HIIT, high intensity interval training*.

**Figure 1:**
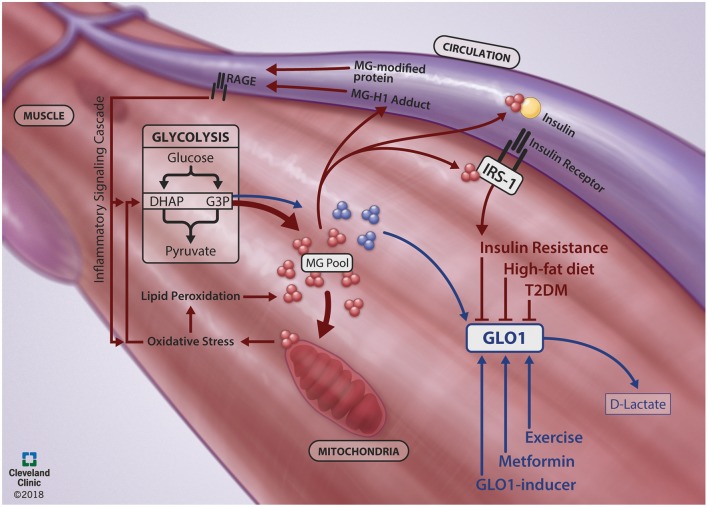
Skeletal muscle MG-GLO1 physiology in the context of health and disease. During healthy physiology (Blue Lines), methyglyoxal (MG) is generated in the skeletal muscle as a spontaneous byproduct from the 3-carbon intermediates (dihydroxyacetone phosphate, DHAP; glyceraldehyde-3-phosphate, G3P) of glycolysis. This MG is efficiently detoxified by glyoxalase-1 (GLO1) to D-lactate. However, GLO1 protein expression is reduced in metabolically impaired states, such as insulin resistance, type 2 diabetes mellitus (T2DM) and during high-fat diet feeding. In concert, MG is generated from glycolysis at an increased rate (Red Lines) and contributes to poor skeletal muscle metabolic health through multiple pathways. Excessive MG modifies mitochondria, contributing to oxidative stress, lipid peroxidation and further generation of MG. Excessive MG also directly inhibits insulin signaling by binding to IRS-1 within the skeletal muscle and by binding to circulating insulin. Further, excessive MG can bind to proteins, presenting in circulation as MG-H1 adducts or MG-modified proteins, which activate the RAGE (receptor for advanced glycation endproducts)-mediated inflammatory signaling cascade. Together, this creates a viscous cycle of MG generation. A potential route to combat dicarbonyl stress is through increasing GLO1 protein expression with targeted therapeutic strategies, such as exercise, metformin and GLO1-inducers. We thank Brandon Stelter and the Center for Medical Art and Photography at the Cleveland Clinic for generating the figure.

Collectively, increasing GLO1 expression may play a protective role when MG generation becomes exacerbated. The protective role of GLO1 to attenuate dicarbonyl stress has potential in maintaining metabolic homeostasis in skeletal muscle. Further clarification of GLO1's role in metabolically compromised cells and elucidation of its regulatory proteins and their stimuli may lead to better avenues for T2DM treatment and prevention. A schematic summary of skeletal muscle MGGLO1 physiology in health and disease is presented in Figure 1.

## Therapeutic strategies for the prevention of dicarbonyl stress

### Scavenging MG and MG-modified proteins

Drugs of the guanidine family are known to function as MG scavengers and bind to the highly reactive MG, preventing MG-induced protein modifications, like MG-H1, in plasma and tissues. The pharmacologic agent, Aminoguanidine, showed much promise, improving laboratory measures predictive of diabetic complications (74). However, lack of clinical outcomes (75) and serious side effects (76) limit its therapeutic application in diabetes. Metformin is the most commonly prescribed medication for T2DM and functions as an MG-scavenger due to its biguanidine structure. Conceptually, MG scavengers may be flawed for applications in skeletal muscle, as MG scavengers require an increase in stoichiometric proportion to MG production; this would require supraphysiologic concentrations of MG-scavengers to scale with the high glucose flux and MG generation within skeletal muscle.

### GLO1 inducer therapy—nutraceutical

GLO1 inducer therapy increases GLO1 protein expression by stimulating Nuclear factor-erythroid 2 p45 subunit-related factor 2 (NRF2). NRF2 is a transcription factor that promotes basal and inducible expression of GLO1 (77, 78) and is implicated in T2DM in both animals and humans (79–81). Recent human trials have shown the effectiveness of trans-resveratrol and hesperetin (tRES/HESP) to increase GLO1 protein expression and activity concomitant with reductions in plasma MG and MG-directed protein modifications (82) via NRF2 signaling (83). This recent work has achieved effective pharmacologic targeting of the NRF2-GLO1-MG axis in human plasma concomitant with clinically relevant improvements in whole body glucose metabolism. This profound effect on glucose control elicited by tRES/HESP therapy are suggestive of effects on highly metabolic tissues, like the liver and skeletal muscle. Future research should investigate the liver and skeletal muscle effects of tRES/HESP therapy on the tissue specific NRF2-GLO1-MG axis. For information on other GLO1 inducer formulations, the reader is directed to a recent review by Rabbani and Thornalley (84).

### GLO1 inducer therapy—aerobic exercise

The relationship between physical activity and T2DM is well known. A sedentary lifestyle contributes to insulin resistance, while increased physical activity improves insulin sensitivity and whole-body glucose and lipid metabolism (85, 86). Chronic aerobic exercise has been shown to have robust benefits on insulin sensitivity, glucose disposal, glycogen content, fatty acid oxidation and metabolic flexibility (87–102). Both acute (103, 104) and chronic aerobic exercise (92, 99, 100) provide these benefits which are driven by adaptations in skeletal muscle (105, 106). Furthermore, individuals at risk for developing T2DM (obese, insulin-resistant individuals) can reduce the risk of progression to T2DM by over 50% with a lifestyle intervention that focuses on reducing calorie intake and increasing exercise (2). At this time, the signaling mechanisms that underline these metabolic improvements are not well understood but may involve regulation of GLO1 such as exercise-induced stimuli increase NRF2 (64), including oxidative stress (107) and AMPK signaling (108). It is reasonable to infer that NRF2 directs upregulation of skeletal muscle *GLO1* gene in response to these or other exercise-mediated skeletal muscle stimuli.

Microarray studies with exercise have reported mixed results where one study found no differences in *GLO1* when comparing three groups of young healthy men; untrained, aerobically trained and resistance trained (109). Yet another gene microarray showed skeletal muscle *GLO1 mRNA* increases with chronic aerobic exercise training in older men (71). Given the known relationship between increasing chronologic age and reductions in GLO1, it is reasonable to assume these older men had reduced *GLO1 mRNA*, however, there was no comparative lean healthy control group in this study. Together, this implicates a potential ceiling effect for aerobic exercise to increase *GLO1* gene expression. Similar results were found measuring GLO1 enzymatic activity with exercise training. Haralambie et al. (50) performed a cross-sectional investigation of GLO1 activity in untrained and trained individuals, showing a trend for a 21% increase in GLO1 activity in the trained cohort. Importantly, the untrained cohort was young, and fit (VO_2max_: *c.a*. 46 ml/kg/min), and may have had optimal levels of GLO1, again providing evidence of a ceiling for GLO1.

Few studies in human skeletal muscle have investigated the effect of exercise on GLO1 protein expression (8, 51, 72, 82). Hoffman et al. showed acute exercise produces a non-significant 21% increase in GLO1 protein expression using large-scale proteomic profiling (51). Using a similar approach, Hussey et al. showed reduced GLO1 protein expression after 4 weeks of exercise training [3 days per week of aerobic training and 2 days per week of high-intensity interval training (HIIT)] in individuals with T2DM (72). The effect of long-term interventions or different training modalities (continuous aerobic vs. HIIT) is still under investigation. Additional research is needed to corroborate these findings that aerobic exercise confers a therapeutic benefit in individuals with reduced skeletal muscle GLO1 gene expression, protein expression, or enzymatic activity.

### Other considerations for future research

- Skeletal muscle GLO1 protein or gene expression data alone may not offer a complete picture without concomitant measures of GLO1 activity and dicarbonyl stress (i.e., MG-modified proteins and AGEs).
- Many MG-modifications on protein may be inert. Understanding which specific proteins acquire altered function and compose the skeletal muscle dicarbonyl proteome will advance the field greatly.
- Skeletal muscle GLO1 is reduced in mice fed a high-fat diet (66), but whether diet or obesity has independent effects on skeletal muscle GLO1 in humans has yet to be determined.
- Much is still unknown about GLO1 regulation. For example, GLO1 can undergo four unique post-translational modifications that inhibit GLO1 activity (110). Future research should aim to elucidate the independent importance of these modifications.
- With regard to exercise, dose response and time course studies in all modes of exercise (aerobic, resistance, interval, etc.) remain to be empirically tested.

## Summary

Elevated dicarbonyl stress is characteristic of T2DM and associated with the development of diabetic complications. Recent research suggests dicarbonyl stress in skeletal muscle may play a causative role the development of insulin resistance and the onset of T2DM. The primary cellular defense against dicarbonyl stress is the glyoxalase enzymatic defense systems controlled by the protein expression and enzymatic activity of GLO1. Novel therapies targeted at inducing GLO1 improve whole body glucose control, indicative of effects in skeletal muscle, and may represent the next line of adjuvant therapy for T2DM. We have recently described aberrant dicarbonyl stress and GLO1 protein expression in the skeletal muscle of individuals with T2DM. Further, we postulate that aerobic exercise training will result in GLO1 gain-of-function through NRF2 mediated pathways. Additional research is warranted to determine the physiologic impact of targeting skeletal muscle GLO1 and dicarbonyl stress for the prevention and treatment of skeletal muscle insulin resistance and T2DM.

## Author contributions

JM conceptualized and wrote this work. JH conceptualized, wrote and edited this work, and provided funding for trainees. JH provided approval for publication of the content and agrees to be accountable for all aspects of the work in ensuring that questions related to the accuracy or integrity of any part of the work are appropriately investigated and resolved.

### Conflict of interest statement

The authors declare that the research was conducted in the absence of any commercial or financial relationships that could be construed as a potential conflict of interest.
